# Unlimited adaptations of muscle fibres to exercise; are you kidding me?

**DOI:** 10.1113/EP092983

**Published:** 2025-07-05

**Authors:** Hans Degens, Paul Hendrickse

**Affiliations:** ^1^ Department of Life Sciences, Institute of Sport Manchester Metropolitan University Manchester UK; ^2^ Institute of Sport Science and Innovations Lithuanian Sports University Kaunas Lithuania; ^3^ Lancaster Medical School Lancaster University Lancaster UK

1

Wouldn't it be great if we could develop unlimited muscle strength and endurance simultaneously? At least in the realms of sport and bodybuilding, it is often asked whether the ability to put on muscle mass is limited (e.g., https://legionathletics.com/muscle‐gain‐calculator/?srsltid=AfmBOor0e08pHwt70fZ3U‐XPFMvYWmHozuio5UrIsTY9K27HOxrbYsjZ). In the context of muscle fibre morphology, the question becomes whether a muscle fibre can have both an unlimited size and oxidative capacity. Our intuition tells us this is impossible; there must be limits to both. In fact, physiologists agree with this intuition and have suggested that there is a trade‐off between the size and oxidative capacity of a muscle fibre, owing to diffusion limitations (Degens, 2012; Van der Laarse et al., 1998). Yet, resistance training of untrained people induced muscle fibre hypertrophy without a reduction in oxidative capacity (Green et al., 1999), and in highly resistance‐trained men endurance training superimposed on their usual training programme did not induce muscle fibre atrophy despite an increase in fibre oxidative capacity (Hendrickse et al., 2021). Is there therefore really a trade‐off and/or limit to the size and oxidative capacity of a fibre? Is it possible that capillary proliferation does break this apparent trade‐off?

Recently, we sought to assess the limits to possible combinations of the size, oxidative capacity and capillary supply to a muscle fibre (Degens et al., 2026). A research paper does not typically allow one to express the excitement when data do fit expectations beyond your wildest dreams. And exactly that happened during the development of that paper. The journey started with the unexpected observation for human muscle fibres of an apparent upper limit for the capillary density of a fibre at a given fibre cross‐sectional area (FCSA), where smaller fibres had a larger maximal capillary density (Degens et al., 2026: fig. 2a). This was, to us, rather curious, because why would the capillary density of a fibre have an upper limit for a given FCSA or why would, at a given capillary density, the fibre size not exceed a certain FCSA (Figure 1a)? Perhaps it was coincidental? However, if such a relationship exists, smaller fibres should have a higher maximal capillary density and larger fibres a lower capillary density.

**FIGURE 1:**
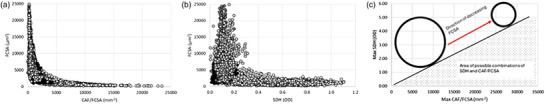
(a, b) Fibre cross‐sectional area (FCSA) versus fibre capillary density [capillaries around a fibre per FCSA (CAF/FCSA)] (a) and oxidative capacity (measured as succinate dehydrogenase optical density (SDH OD) (b) of muscle fibres from mice (open circles), recreationally active humans (black circles) and fibres from highly resistance‐trained men before (dark grey circles) and after (light grey circles) 10 weeks of superimposed endurance training. Each data point represents an individual muscle fibre. (c) The relationship between the maximal fibre oxidative capacity (SDH OD) and the maximal number of capillaries around a fibre per fibre cross‐sectional area (CAF/FCSA).

Muscle fibres from mice are about half the size of human fibres, and to our surprise, we found a similar relationship in the smaller mouse muscle fibres. When we combined the human and mouse fibres, it matched our expectation almost perfectly; a continuous curvilinear relationship appeared between maximal capillary density at a given FCSA, independent of muscle (soleus, extensor digitorum longus, diaphragm or vastus lateralis) or species of origin (Degens et al., 2026: fig. 2c)! We were exuberant and wondered whether this relationship would also apply for extremely large fibres.

Again, we were in the fortunate circumstance that we had data from highly resistance‐trained athletes, who had fibres almost double the size of normal human fibres. When the combined data from mice, humans and highly resistance‐trained men appeared on the graph, we were flabbergasted, because there was a continuation of the curvilinear relationship between the maximal capillary density of a fibre and FCSA (Degens et al., 2026: fig. 2d). It thus appears to be a fundamental feature, independent of species and muscle of origin. In line with the suggested trade‐off between fibre size and oxidative capacity, we also found a curvilinear relationship between the maximal oxidative capacity and FCSA of a fibre (Degens et al., 2026: fig. 5c) and vice versa (Figure 1b), again independent of species and muscle origin.

We proposed in our paper that the inverse relationship between maximal FCSA and maximal oxidative capacity is indeed a reflection of diffusion limitations. Yet, it has been argued that ‘typically metabolic processes in muscle fibres are largely reaction controlled and not greatly limited by diffusion limitation’ (Kinsey et al., 2011). We suggest that this is the case as long as the fibres stay below the maximal oxidative capacity for a given FCSA, reflected by the dotted area in fig. 7 of Degens et al. (2026); reproduced here as Figure 1c. Indeed, estimates (Kinsey et al., 2007) show that for a juvenile fibre of the blue crab (diameter < 80 µm) (Hardy et al., 2009), metabolites travel half the distance between homogeneously distributed mitochondria in <20 ms, whereas in adult fibres (>600 µm in diameter) the time required to travel half the distance from the peripheral mitochondria on one side to the opposite side of the fibre is 7.5 min, clearly placing a limit on aerobic metabolism and even limiting recovery from a short anaerobic burst of activity. In this context, it is interesting that it has been suggested that many fibres are working at the edge of diffusion limitation for oxygen and metabolites, and prevent diffusion limitation by cell division (Kinsey et al., 2011), whereas in the enormous aerobic fibres of the blue crab this problem is solved by compartmentalisation and highly perfused subdivisions of individual fibres (Hardy et al., 2009).

In adult mammals, there is no evidence of compartmentalisation of large fibres, nor much evidence for fibre hyperplasia. This then begs the question of what mechanisms underlie the maximal attainable combination of capillary supply, oxidative capacity and size of a fibre we observed, independent of muscle or species origin?

We previously suggested that muscle fibre size limitations might be attributable to constraints to nuclear domain size, bone and joint strength, pennation angle (where an increase in pennation angle beyond 45° owing to fibre hypertrophy does not further increase the force‐generating capacity of the muscle) and tendon strength (Degens, 2012). In the new paper (Degens et al., 2026), we discussed how the limitations of the size, oxidative capacity and capillary supply of a muscle fibre are attributable to diffusion limitations and physical constraints of capillary position. We did not discuss, however, what mechanisms underlie the limits of oxidative capacity, size and capillary supply to a fibre. Although it has been acknowledged that intracellular transport distances (Miettinen et al., 2017) and genetics impose a physical limitation on cell size, supported by plateauing of exercise‐induced hypertrophy and a progressive attenuation of exercise‐induced anabolic pathways (Kataoka et al., 2024), it raises the question of how these pathways become less and less stimulated even when progressively increasing the training load. Although there is too little room to discuss this extensively, here we propose a working framework.

Shear stress and hypoxia elicit angiogenesis, whereby capillary proliferation reduces both endothelial shear stress and muscle tissue hypoxia. It is possible that beyond a certain capillary density, the shear stress per capillary drops below the threshold to stimulate further angiogenesis. Although hypoxia will also stimulate angiogenesis, the absence of further reductions of the diffusion distances by having more than two capillaries per fibre might, in large fibres, continue to result in activity‐induced hypoxia (even if many more capillaries were added) that then stimulates glycolytic metabolism and suppresses aerobic metabolism, perhaps via hypoxia‐inducible factor‐1α (Hoppeler & Vogt, 2001). We suggest that the reduced reliance on aerobic metabolism, in turn, reduces the hypoxic stimulus and results ultimately in a new steady state where fibre size, oxidative capacity and capillary supply to a fibre are matched.

In addition to mechanisms related to diffusion limitations of oxygen and metabolites, one can imagine that in a pennate muscle, any further increase in fibre size beyond that resulting in a pennation angle of 45° will make it impossible to increase the mechanical tension on individual muscle fibres further, hence no further increase in fibre stress (an important stimulus for anabolic signalling pathways; Erskine & Degens, 2013) will be possible. This then causes cessation of further muscle fibre hypertrophy. We acknowledge that these suggestions are speculative, but given the limits to muscle fibre size, it is perhaps interesting to test the validity of this framework in future studies.

Finally, the maximal combinations of size, oxidative capacity and capillary supply of a fibre were described only in fibres from healthy people and animals, and not in atrophied fibres, fibres from organisms exposed to hypoxia (as in altitude) or in disease states. It might well be that in such cases we find different relationships. Some indication for possible altered maximal combinations is the hypoxia‐induced atrophy accompanied by opposite changes in oxidative capacity and capillary supply to a fibre in the deep and superficial region of the rat plantaris muscle (Wüst et al., 2009). It therefore remains to be seen whether in hypoxia, disease states or pharmaceutical interventions (with or without extreme training programmes) similar situations apply. Nevertheless, the data in our paper, for the first time, show evidence for limits to the size, oxidative capacity and capillary supply to a fibre attributable to both diffusion limitations and physical constraints.

## AUTHOR CONTRIBUTIONS

Study design: Hans Degens. Writingoriginal draft preparation: Hans Degens. Writing and final editing: All authors contributed to writing‐review and editing the manuscript. All authors have read and approved the final version of this manuscript and agree to be accountable for all aspects of the work in ensuring that questions related to the accuracy or integrity of any part of the work are appropriately investigated and resolved. All persons designated as authors qualify for authorship, and all those who qualify for authorship are listed.

### CONFLICT OF INTEREST

The authors declare no conflicts of interest.

### FUNDING INFORMATION

None.
